# Dynamic Optimization and Heuristics Based Online Coverage Path Planning in 3D Environment for UAVs

**DOI:** 10.3390/s21041108

**Published:** 2021-02-05

**Authors:** Aurelio G. Melo, Milena F. Pinto, Andre L. M. Marcato, Leonardo M. Honório, Fabrício O. Coelho

**Affiliations:** 1 Department of Electrical Engineering, Federal University of Juiz de Fora, Juiz de Fora 36036-900, Brazil; aurelio.melo@engenharia.ufjf.br (A.G.M.); andre.marcato@engenharia.ufjf.br (A.L.M.M.); fabricio.coelho2010@engenharia.ufjf.br (F.O.C.); 2 Department of Electronics Engineering, Federal Center for Technological Education of Rio de Janeiro, Rio de Janeiro 20271-110, Brazil; milena.pinto@cefet-rj.br

**Keywords:** coverage path planning, 3D path planning, waypoint graph, mapping, navigation, UAVs

## Abstract

Path planning is one of the most important issues in the robotics field, being applied in many domains ranging from aerospace technology and military tasks to manufacturing and agriculture. Path planning is a branch of autonomous navigation. In autonomous navigation, dynamic decisions about the path have to be taken while the robot moves towards its goal. Among the navigation area, an important class of problems is Coverage Path Planning (CPP). The CPP technique is associated with determining a collision-free path that passes through all viewpoints in a specific area. This paper presents a method to perform CPP in 3D environment for Unmanned Aerial Vehicles (UAVs) applications, namely 3D dynamic for CPP applications (3DD-CPP). The proposed method can be deployed in an unknown environment through a combination of linear optimization and heuristics. A model to estimate cost matrices accounting for UAV power usage is proposed and evaluated for a few different flight speeds. As linear optimization methods can be computationally demanding to be used on-board a UAV, this work also proposes a distributed execution of the algorithm through fog-edge computing. Results showed that 3DD-CPP had a good performance in both local execution and fog-edge for different simulated scenarios. The proposed heuristic is capable of re-optimization, enabling execution in environments with local knowledge of the environments.

## 1. Introduction

Path planning is one of the essential issues in the robotics field. According to Reference [1], it refers to find an optimal route for an object that moves from a start point to a final one. Path planning is applied in many domains ranging from aerospace technology and military tasks to manufacturing, and agriculture [2,3,4]. Path planning is a branch of autonomous navigation problem. In autonomous navigation, dynamic decisions about the path have to be taken simultaneously while the robot moves towards its goal [5]. Many works have been developed to improve the many branches of path planning and autonomous navigation, as in Reference [6,7,8].

Over the past years, autonomous navigation and path planning, along with Unmanned Aerial Vehicles (UAVs), have been facing unprecedented growth [9,10]. They rely on the fact that they have high mobility, scalability, and flexibility to perform missions with short path length, maximum roll angle, and fast speed. UAVs are applied to various areas, such as search and rescue [11], agriculture [12], and inspection [13,14], among others. One of the main goals in applications with UAV is to build intelligent systems capable of performing different tasks without human intervention [15]. For instance, the technical requirements for visual inspection of large structures, such as slopes and damns, create a perfect task for the application of UAVs [16].

Among the navigation area, an important class is Coverage Path Planning (CPP). The CPP technique is associated with determining a path that passes through all viewpoints in specific areas and the collision-free paths to travel to each viewpoint. This kind of algorithm must be efficient, as well as able to provide sufficient information for a later processing stage of analysis [17]. Within this research scope, the CPP problem is about planning waypoints to inspect complex surfaces and the collision-free path connecting those points in an optimized way.

Path planning can be performed online, offline, or in a combination of both according to determining application requirements. Many previous works focused on known environments, such as Reference [18], where the authors presented a multi-heuristic A* algorithm for solving a path planning problem for a tethered mobile robot. However, the algorithm could not be handled in dynamic scenarios. In the last few years, intelligent methods have been proposed for robot planning strategy in dynamic scenarios [19]. In unknown dynamic environments, the robotic system must obtain distance information of its current position state and obstacles and then generate a trajectory in an online way. In the last few years, online path planning in unknown environments has attracted a lot of interest [20].

Three-dimensional path planning techniques are crucial for the navigation of UAVs in complex and highly dynamic environments. Besides, it faces many uncertainties. For instance, optimality is one of the major challenges for UAVs’ path planning, and it defines that the system should be time, cost, and energy-efficient. Other factors are path length, robustness, and collision avoidance [21,22]. In this sense, a standard solution is the use of two different approaches, that is, a global one responsible for finding solutions to the complex problem and a local solution for the collision avoidance of dynamic obstacles.

In the last few years, many methods have been developed for 3D environments, such as Visibility Graph [23], Rapidly-exploring Random Tree [24,25], and Probabilistic Roadmap [26]. In relation to optimal search algorithms, it can be cited A* [27], D* [28], Dijkstra’s algorithm [29], and bio-inspired algorithms [16], among others. Many of those methods that rely on heuristics may not find optimal paths in general. Besides, it is not possible to use the linear optimization model directly in an unknown environment. This is related to the fact that the linear model has no way of performing planning for the unknown parts of the space [21], requiring additional mechanisms to deal with the addition or removal of nodes to be visited, avoiding a full optimization of point set from scratch. This opens a space for the combination of optimization methods with heuristics to search for sub-optimal paths online while the task is performed.

As can be seen, path planning is a very active research topic. Despite the many methods and challenges, a constant among them is the goal of finding the possible best solution in a minimum amount of time and with less computational cost possible [30,31]. Note that the definition of good solution changes following application requirements. For example, some applications may require the shortest path, while others may require less energy consumption.

1. Optimization type: The methodologies for path planning can be subdivided among few groups related to their characteristics. Here, we will classify among machine learning, mathematical models and bio-inspired.
2. Energy efficiency: Being energy efficient will be defined here as the path that takes less battery or fuel for fulfilling the desired trajectory goals.
3. Time efficiency: Time efficiency means that the minimum time or total distance to fulfill all trajectory goals.
4. Online: Online is class of methods that are executed while the robot move towards its goal. Authors evaluated as online any method that can be used on-board the UAV to find paths to trajectory goals while in flight.
5. Optimality: This feature means that the path can be optimal or sub-optimal in regards to time, distance, or energy efficiency.
6. Consider local conditions: This feature means that the method can consider local environmental conditions, such as wind and payload.

Table 1 evaluates some works in the state-of-the-art for those features along with the proposed method 3D dynamic for CPP applications (3DD-CPP). The symbols used in the table are: ✓: has feature, ×: does not have feature, −: not described. The references used in the table were selected looking for the higher relevance in the database of SAGE, IEEE, and Elsevier journals and conferences using the metric of Equation (1).

(1) $$\begin{array}{c} number\_citations-2\;\cdot \;years\_from\_publication^{2}. \end{array}$$

In the literature, there exist some CPP methods that are closely related to the proposed CPP method. In Reference [22], the authors proposed the usage of sweeping pasterns to define points to be visited, and they used ant colony optimization to find the optimal interconnection between pasterns. A similar problem can be seen in Reference [38]. In their work, a Genetic Algorithm (GA) was used to determine paths in order to find a viable CPP trajectory. As can be seen, there is still space for improvement and proposition of new methods despite the many methods in the state-of-the-art. For instance, mathematical models have other advantages that are predictable behavior and deterministic response. For instance, many works do not explicitly define a model to estimate robot energy usage, allowing this factor to be included in the modeling. A few of the compared works also evaluated the required characteristics of waypoints positions on inspection applications. This aspect of the environment is evaluated when defining the proposed model.

In order to improve from the literature, this research presents a model to estimate UAV energy usage while moving through space. This model can consider both UAV and environmental characteristics, such as wind. This work also lays ground into the ideal way to sample inspection waypoints in space according to the desired performance. Therefore, this research uses a mathematical model combined with update heuristics to build the optimization method. A concern of the work is the computational viability of the technique. This is especially important for mathematical methods where a high computational power may be required. In this sense, parallel execution in a distributed environment is evaluated. This is an innovative aspect of this work, where part of the code is executed onboard the robot and part of it in the fog close to the robot in an edge-fog scheme [39].

These kinds of techniques allow a UAV to explore a given area without requiring previous full models of the area. This is advantageous for many applications, such as inspection of dams and slopes [40]; and agricultural applications, such as collecting analytical data about soil, vegetation, or animal life [41], or intervening by precision application of liquid fertilizers, pesticides, or herbicides [42], among others. Other possible applications include forest management [43] and surveillance [44].

### 1.1. Main Contributions

Besides the fact that there are many practical methods to perform CPP, there is still space for improvements considering UAV or environment characteristics in different ways that can result in optimal performances and safe planning. The main contribution of this work is to present a method that can be applied to produce optimal results in known environments or sub-optimal ones in a partially known environment. The contributions of this work can be summarized as follows:

- Present an optimization method to perform CPP for aerial 3D applications.
- Evaluate heuristics to allow the deployment of the method in unknown locations.
- Evaluate a strategy to estimate cost matrices allowing optimization of energy usage.
- Evaluate the effectiveness of the proposed CPP method by comparing the simulation results with prior research.

### 1.2. Organization

The rest of this work is organized as follows. In Section 2, Proposed Methodology, a detailed analysis of 3DD-CPP coverage path planning is provided. Section 3, Simulation, Results, and Discussions, presents the results and discussions comparing the 3DD-CPP with the state-of-the-art algorithms in coverage path planning. The conclusions and final remarks are conducted in the Section 4, Conclusions and Future Work.

## 2. Proposed Methodology

**Figure 1:**
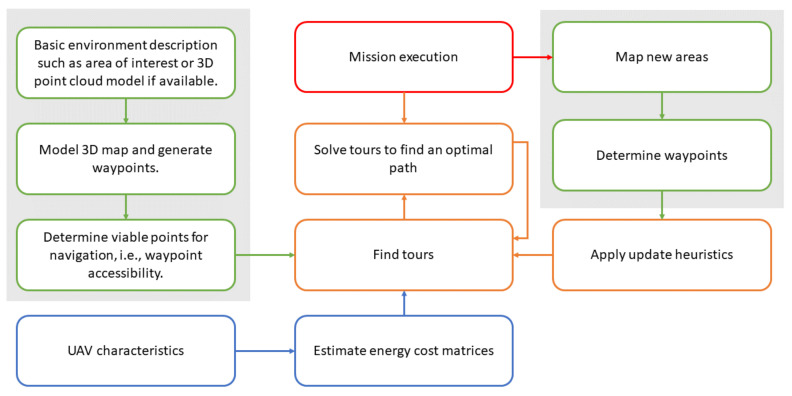
Overview of the proposed method.

Figure 1 illustrates a general overview of how the proposed method would be integrated. The first steps would consist of gathering information about the environment to be inspected. This means that it is necessary to define at least the general shape and size of the area to be inspected. And in the best case, obtain previous 3D models of the area. If 3D models are available, they have to be processed to determine the waypoints. In this work, the authors have not focused on this step. However, Section 3.3, Real Point Cloud, provides some insights into this process. These steps are with a background in light grey as they were not detailed described.

In another branch of Figure 1 represented by the blue boxes, the UAV characteristics are evaluated. The UAV model is used to estimate the energy cost for moving in each direction at a given speed. Speed and energy usage have a trade-off that is currently not directly considered in the model. Thus, if the user desires to optimize the time to complete the task, he/she can increase the UAV speed and update the cost matrices to account for the new energy requirements. At this point, the optimization method can be applied, represented by the orange boxes. The path outputs will be fed into the execution algorithm responsible for carrying out the mission. If new waypoints are discovered, the update heuristics are applied, and the optimization methods take over again.

Several concepts have to be introduced in order to delineate the proposed methodology and environment. Therefore, the next subsections will define models to represent the many elements of the issue. Section 2.1, Space Representation, defines the space representation approach used in this work. Section 2.2, Optimization Methodology and Section 2.3 Energy Cost Estimation, present the details about the used models. Finally, Section 2.4, Optimization Model, compiles these information into a single model.

### 2.1. Space Representation

A proper space representation of the environment is defined to allow the modeling of the proposed problem. Note that many classic path planning algorithms use a grid-based approach [37]. In this approach, the 2D space is discretized into grids where the robot can exist. This approach simplifies the problem once it allows the robot movement to be organized in structures, such as lists, trees, and graphs.

**Figure 2:**
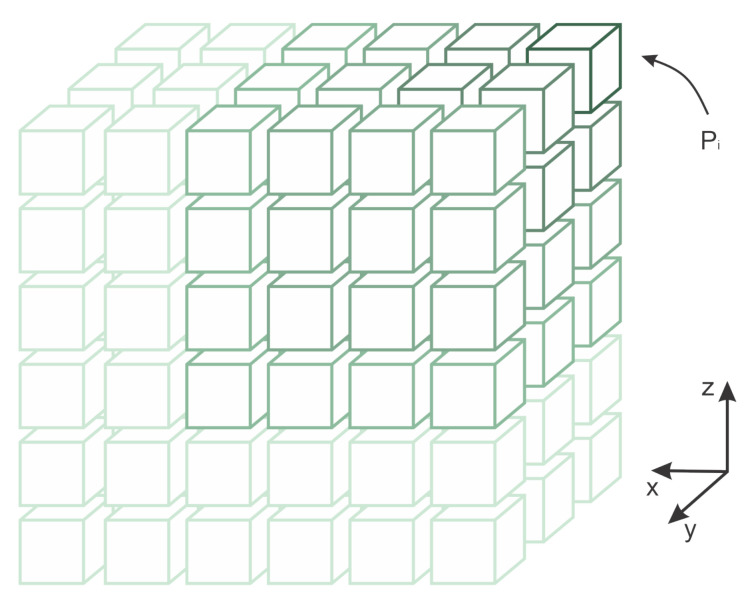
Cellular representation of the space.

In this work, a similar approach is presented, and it is adopted. The grid has to be replaced by a cell to represent a 3D space, as represented in Figure 2. Then, the space will be represented by a 3D matrix. The three elements represent the position, i.e., $p_{x},\;p_{y},\;p_{z}$.

Any measurement in the real world is continuous by nature. However, in this representation, the possible positions are assumed to be not continuous. In this sense, the real-world is quantized concerning the surface cell size. This can be noticed in Figure 2. In this figure, the space becomes quantized in its dimensions. This quantization is an advantage in many applications once it simplifies the surface structures and smoothes the details, simplifying the processing.

Authors believe that this structure is also advantageous in the proposed application. The quantized surface can be optimally defined to meet image inspection requirements. The size will be dependent on several factors, such as camera data (field of view, sensor size, and resolution) and inspection requirements, such as image overlap. In this type of application, a given image overlap is required for structure from motion or mapping techniques.

**Figure 3:**
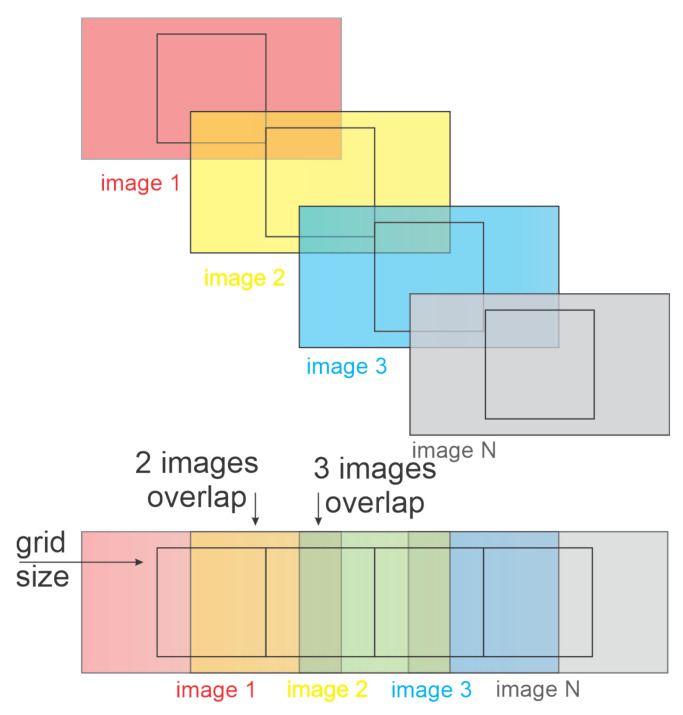
Image overlap representation.

Figure 3 has an image overlap representation in relationship with the quantized surface representation. Note that the grid size represents the quantized surface unit. Smaller grid sizes will increase the image overlap. Image 1, in the figure, is represented in red, and image 2 in yellow. The overlap area between them is in light brown. The same can be observed in image 2 (in yellow) and image 3 (in blue), where the intersection between them is represented in green.

Note, in Figure 3, that the forward movement creates an overlap between the images in one direction. A second direction is a lateral overlap created by the distance among the inspection lines. It is possible to model the overlap considering $dimg_{ij}$ as the distance among centers of the image *i* and *j*, and *H* the distance between the camera sensor and the surface. Equation (2) exemplifies this overlap in relation to the camera parameter $cam_{conf}$. The parameter $cam_{conf}$ represents the relation between the camera focal length and its sensor width size.

(2) $$\begin{array}{c} overlap=1-\frac{dimg_{ij}}{H}\cdot cam_{conf}. \end{array}$$

**Figure 4:**
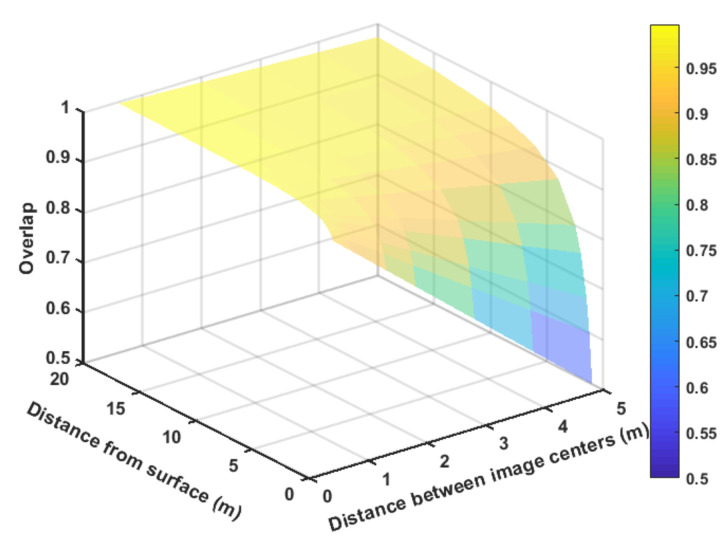
Simulated overlap surface.

Figure 4 shows a representation of the curve generated by Equation (2). This curve was generated to a fixed value of $cam_{conf}$ equals to 0.1. The curve allows a generic visualization of the overlap behavior to the user. The values in the graph were selected to represent the better general behavior. However, overlaps higher than 0.2 can be acceptable for many applications.

When selecting an overlap value, the environment model can be affected directly, which means that, by increasing the image overlap, the surface dimensions’ size will be directly reduced in the environment model. In this sense, these parameters do not need to be carried out to the optimization model, and nevertheless, they will be enforced once this position will be planned as part of the trajectory. Thus, this approach simplifies the proposed optimization model.

A second parameter that has to be taken into account is the ground sample distance (GSD). This is the amount of area that represents a single pixels. This measure provides a general view of the final image quality. GSD can be calculated using Equation (3), where $w_{sensor}$ is the camera sensor width, and $\theta_{sensor}$ is the angle between sensor and the surface, $p_{size}$ is the sensor pixel size, i.e., $p_{size}=\frac{sensor\;width}{vertical\;resolution}$. This formula assumes that horizontal and vertical resolutions and sensor size are proportional; if this is not the case, the horizontal and vertical sampling distances will be different. Note that $\cos(\theta_{sensor})$ is equal to 1 when they are orthogonal, which is the ideal situation.

(3) $$\begin{array}{c} GSD\;=\;\frac{p_{size}\cdot \;w_{sensor}}{cam_{conf}}\;\cdot \;H\;\cdot \;\cos\left(\theta_{sensor}\right). \end{array}$$

**Figure 5:**
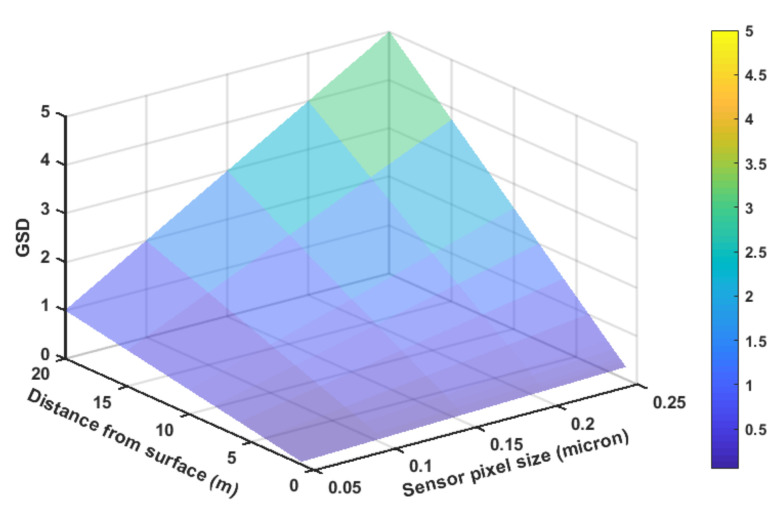
Simulated ground sample distance (GSD) for various sensors’ pixel size and surface distance.

Figure 5 shows a GSD representation for a fixed camera resolution and width. Notice that larger GSD values mean less picture quality overall. By combining a maximum desired value of GSD and overlap, it is possible to determine the maximum distance from the surface and forward step when determining the UAV waypoints.

**Figure 6:**
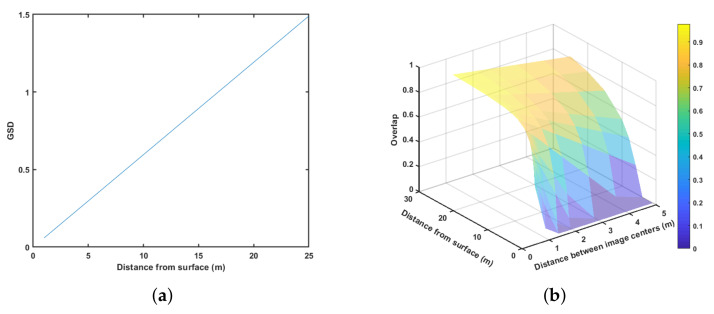
DJI Phantom 4 camera quality in relation to the waypoint parameters. (**a**) GSD vs. distance. (**b**) Overlap in relation to distance of surface and distance between image centers.

As an example, using the camera configurations for DJI Phantom 4, it is possible to estimate these curves, as detailed in Figure 6. Note that a possible higher resolution will always be desired once it increases image quality. As such, this parameter was not included in the graph, resulting in a single curve as in Figure 6a. By selecting the minimum GSD, a given maximum distance from the object is defined. From this point, the distance between image centers will be calculated from the minimum overlap possible, as in Figure 6b.

From these two parameters, it is now possible to estimate the number of waypoints for a given area. This will also determine the sizes of each cell in the surface representation. This process is somewhat deterministic once image quality requirements usually do not change during a given mission. If they do so, the recalculation of waypoints can be performed at the outside path optimization loop. Note that, in the case that multiple UAVs with different camera configurations are used, they can also apply these methods independently. As a result, each aircraft may require a certain number of waypoints concerning its camera specifications.

### 2.2. Optimization Methodology

**Figure 7:**
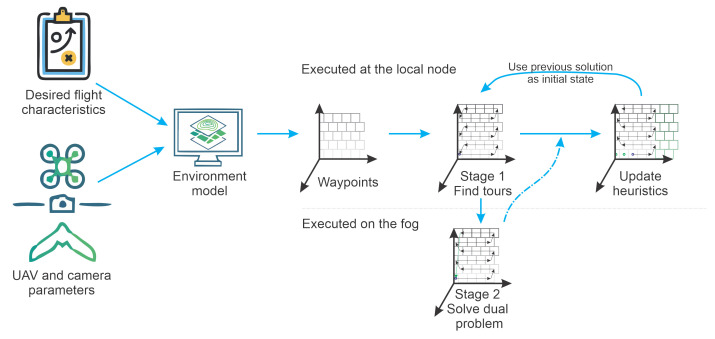
Optimization model workflow.

From the representation showed in Section 2.1, Space Representation, it is possible to build the 3DD-CPP methodology to solve the planning. Figure 7 illustrates the proposed flow. First, the current environment model uses the robot to find optimal tours using optimization. The tour restrictions are fed into a fog/cloud machine. The dual optimization problem will be solved using dynamic programming at the remote machine, finding cutting planes. While this happens, the inspection starts based on the initial solutions. The assumption here is that these stages will happen fast enough (less than a few seconds) to avoid any relevant parts of the mission being executed. An update heuristics will then combine the incoming information from the fog/cloud nodes with the local information about the mission. The local information comprehends newly found inspection areas, already searched areas, and robot position.

**Figure 8:**
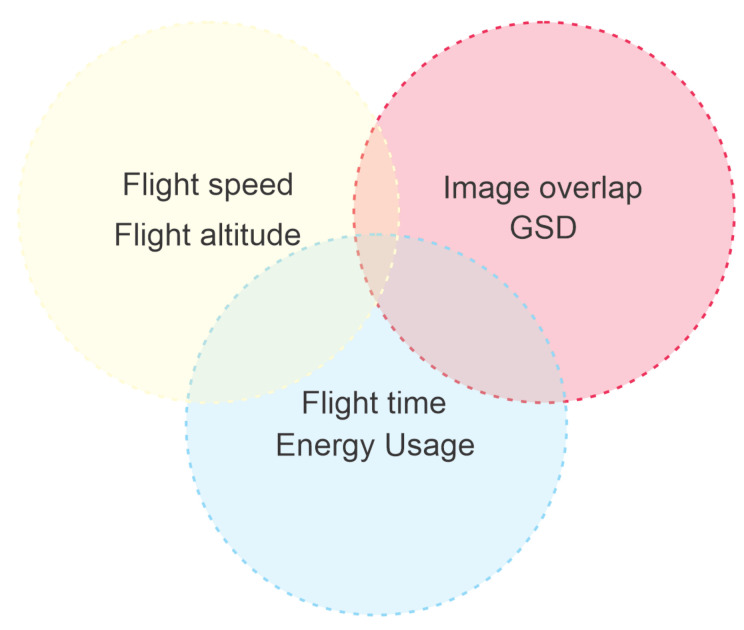
Conflicting optimization goals.

The optimization model used in this work will have to deal with a few conflicting goals. Among those, it should optimize energy usage and flight time while still maintaining image parameters, such as GSD and overlap. Many other parameters are also related to this problem, such as flight speed, distance from the target, image sensor characteristics, etc. Figure 8 presents a graphical representation of the conflicting optimization goals.

### 2.3. Energy Cost Estimation

A mathematical model was devised to estimate the UAV’s energy usage to perform different types of movements. The first essential requirement to be observed is the required torque amount to hover and then move forward. As a simplification, the necessary force to hover at a fixed height and position is equivalent to the UAV weight. When moving at a constant height, an additional force is required to overcome the generated drag. The weight lifting force given by the $mass_{UAV}$ multiplied by gravity *g* and drag force $F_{drag}$ are presented combined in Equation (4). Note that other forces are involved. However, they are minor, and, for simplification, they will be disregarded. The drag is related to the UAV speed $UAV_{speed}$, the medium density $\rho_{air}$, drag coefficient $C_{D}$, and the cross-sectional area A. Equation (5) presents the classical formulation [45,46].

(4) $$\begin{array}{c} Thrust_{Total}=mass_{UAV}\cdot g+F_{drag}, \end{array}$$

(5) $$\begin{array}{c} F_{drag}=\frac{1}{2}\cdot \rho_{air}\cdot C_{D}\cdot UAV_{speed}^{2}\cdot A. \end{array}$$

From the thrust required in Equation (4), it is possible to establish the power requirement from motor dynamics. To allow easy correlation with UAVs’ characteristics, the model will use typical motor data. Table 2 presents the characteristics for the T-Motor MT-3515, as provided by the manufacturer [47]. The use of this table means that the aerodynamic and constructive parameters are comprised of a simple power-thrust relationship.

**Figure 9:**
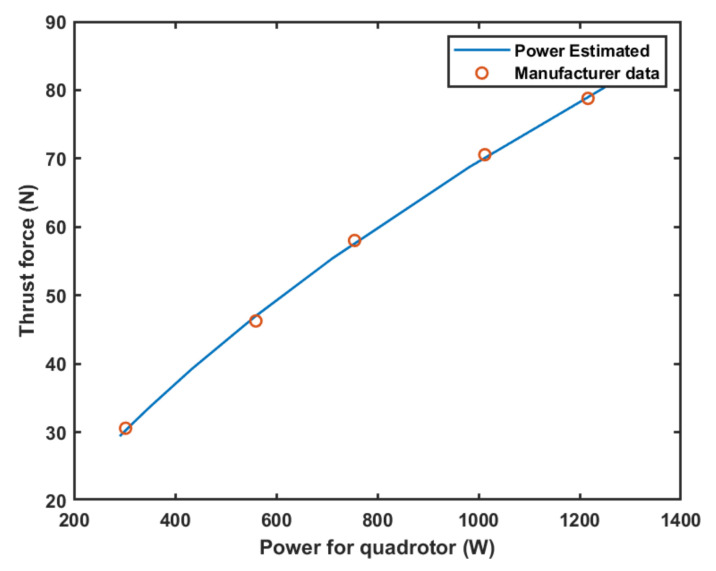
Power-thrust in accordance with Table 2.

Using the data from Table 2, it is possible to estimate the power-thrust relation for every value of thrust by performing a quadratic fit of the data. This process adjust will result in an equation $P_{quad}=c_{2}\cdot Thrust_{Total}^{2}+c_{1}\cdot Thrust_{Total}+c_{0}$, where the coefficients $c_{n}$ are determined by the polynomial fit process. Figure 9 shows this result for a quadrotor using the motor data from Table 2.

The curve in Figure 9 allows easy estimation of flight times and distances for a given battery. However, the estimation of the amount of energy provided by a given battery $\left(Batt_{en}W/h\right)$ is complex due to several different factors, such as discharge curves and minimal voltage, among others. In this sense, this work assumes directly the amount of available power instead of using battery voltages and current to do so. Flight time and remaining flight distances can be estimated as shown in Equations (6) and (7). Those equations are important once they allow mission feasibility analysis, that is, the remaining flight time and the possible max distance covered by the UAV at a given height.

(6) $$\begin{array}{c} Flight_{time}=\frac{Batt_{en}}{P_{quad}}, \end{array}$$

(7) $$\begin{array}{c} Flight_{dist}=UAV_{speed}\cdot \frac{Batt_{en}}{P_{quad}.} \end{array}$$

**Figure 10:**
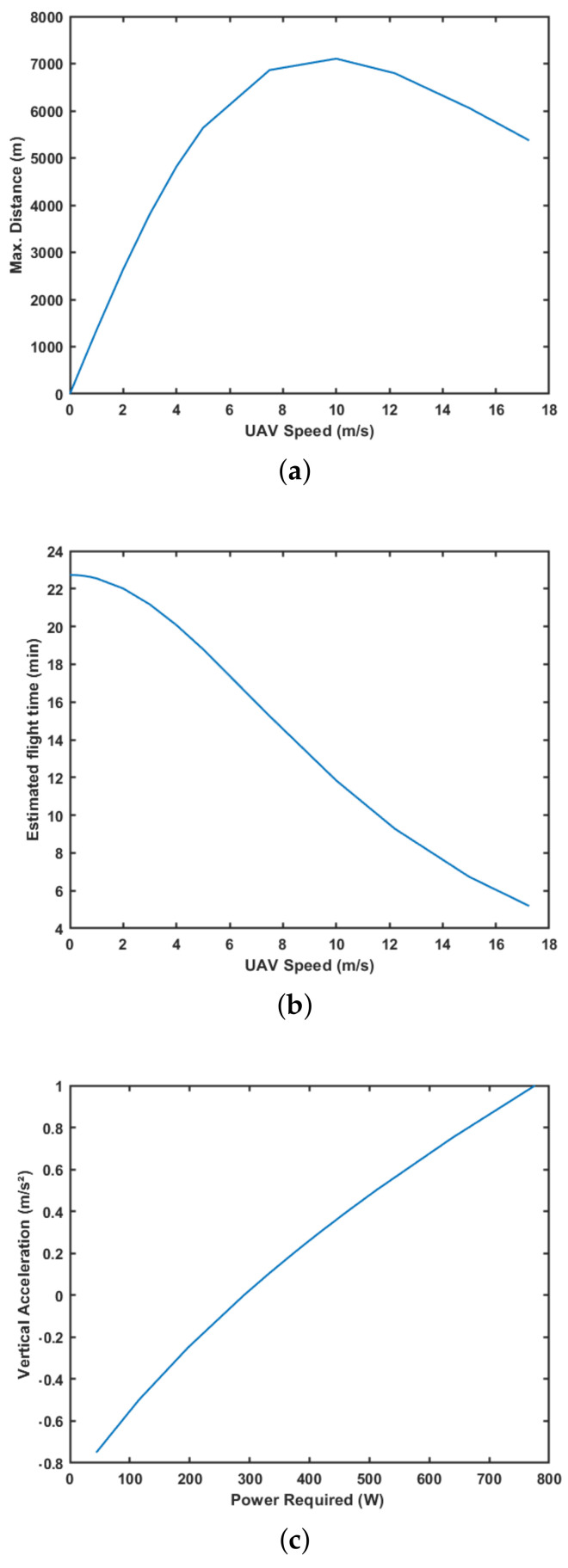
Unmanned Aerial Vehicle (UAV) flight characteristics for a 110 W/h battery. (**a**) Maximum distance traveled. (**b**) Maximum flight time. (**c**) Power required to accelerate vertically.

The model showed before does not take into account changes in the UAV height. It only accounts the forward motion evaluated by the drag force $F_{drag}$. Therefore, a little modification in Equation (4) can be performed including the vertical acceleration $UAV_{vaccel}$ along with gravity resulting in $Thrust_{Total}=mass_{UAV}\cdot \left(g\cdot UAV_{vaccel}\right)+F_{drag}$. Considering a 110W/h battery and variation speed and vertical acceleration, it is possible to estimate UAV characteristics. Figure 10a,b present the maximum distance and flight time for a set of speeds, respectively. Figure 10c shows the power required for a different number of vertical accelerations.

The model showed in this section can be used to produce a rough energy consumption estimation for the principal types of movements that a UAV has to perform. Using this model, one can calculate forces required by horizontal and vertical movements that have to be combined independently to form path estimations. Note that external forces, such as wind, can be taken into consideration by applying them as a type of drag when faced in the horizontal direction. However, it is important to emphasize that this is an approximation. As many of the parameters used are difficult to estimate, practical applications can obtain these estimative from real experimentation using a UAV.

### 2.4. Optimization Model

The selected representation is designed to ensure a good solution with enough detail to fulfill inspection requirements. This representation also allows a feasible optimal solution to be searched. This work uses the properties of the space representation selected to build the optimization problem. In order to understand the process, we have to define the optimization problem as follows.

**Figure 11:**
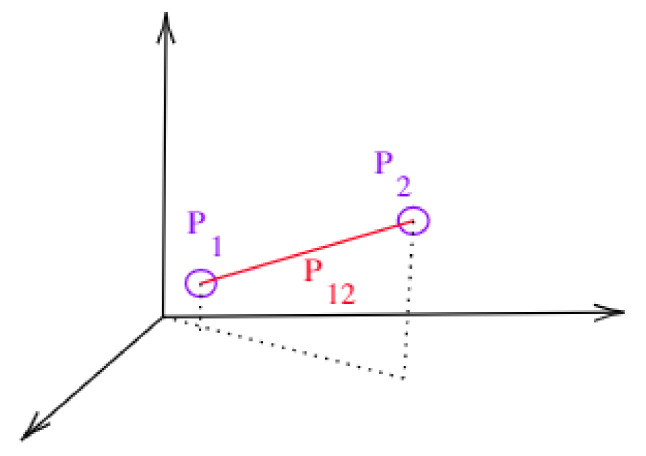
Simplified representation of the coordinate system.

Each inspection position $p_{i}$ has a set of three coordinates $\left\{pos_{X},pos_{Y},pos_{Z}\right\}$ and a surface orientation $\left\{s_{o}\right\}$ encoded as shown in previous section. As explained, the surface orientation is not directly used here for path planning. However, this information is important in future works to allow proper orientation of the camera onboard the UAV. Then, each position is enumerated sequentially. An incidence matrix $p_{ij}$ will be formed representing the path between two points $p_{i}$ and $p_{j}$. The cost of moving between points $p_{i}$ and $p_{j}$ can be calculated using their real positions. This representation simplified the planning for an n-dimensional problem, where more dimensions can simply be encoded in the coordinate set. Figure 11 shows a simplified representation.

A second cost matrix $c_{ij}$ will represent the energy cost of moving from one camera position to the other. This cost matrix will be estimated using the models presented in Section 2.3. The optimization problem is presented in Equation (8). Note, in this problem, that there are no restrictions related to the path size to relax the solution. In addition, note that a more relaxed version can also be builded, allowing the sum of arches connected to each node being larger than 2. The output of this stage is a matrix where all paths from the point *i* to *j* given by $p_{ij}$ belonging to the solution are set as 1, while others are set to 0.

The cost matrix $c_{ij}$, built-in Section 2.3, represents the energy required to move the UAV. When deploying this method to an online user, this matrix can be updated along the flight using a heuristic method. This allows the local conditions to be accounted for. For example, suppose the wind changes and the UAV starts to face resistance. In that case, this matrix can be re-estimated, and the model will be solved using the previous solution as an initial condition.

(8) $$\begin{array}{cc} \min\sum_{i,j=1}^{n} & p_{ij}\cdot c_{ij}\\ s.t & \sum_{j=i}^{n}p_{ij}=2\;\;\forall \;points\;i\;|\;i\neq j\\ \; & 0\;≦p_{ij}≦\;1\;\;\forall \;i,j \end{array}.$$

Note that $p_{ij}$ represents a path between the points *i* and *j*. In this sense, the restriction $\sum_{j=i}^{n}p_{ij}=2\;\;\forall \;points\;i\;|\;i\neq j$ will ensure that, for each node, only two paths will be selected. These two paths will represent an incoming and one outcoming path from each node in the real world. This solution to the problem generates tours that presents feasible solutions and can be denominated $p_{ij}^{*}$. However, these solutions may not form complete paths. In this sense, a dual optimization problem will be solved using dynamic programming. The dual problem is designed to maximize paths sizes, that is, to find sub-tours and add restrictions to the optimization problem associated to these tours.

Consider a given point $pt\in p_{ij}$, and a subset $\Theta \;\subset \;p_{ij}|i<j\;\forall \;i,i<n-1\;$, where n is the number of arches $p_{ij}$. This subset Θ defines the possible route taken by each point. For a give point pt, we can define a set that contains the sub-tour that passes through these vertices as τ. Now, a second subset can be defined as $\vartheta_{i}|\vartheta_{i}=1\;\;if\;\;i\in \tau ,\;i\neq pt,\;\vartheta_{pt}=1\;$. This last set determines all points that a part of a given sub-tour that contains pt. This allows us to build the maximization dual problem as follows in Equation (9).

(9) $$\begin{array}{cc} max & \left(\sum_{i,j\;in\;\Theta }p_{ij}^{*}\;\cdot \;d_{i,j}-\sum_{i=1}^{n}\vartheta_{i}\right)\\ s.t & \;\;d_{i,j}\geq \vartheta_{i}+\vartheta_{j}-1\\ \; & d_{i,j}\leq \vartheta_{j}r\\ \; & d_{i,j}\leq \vartheta_{i} \end{array},$$

where $d_{i,i}$ represents a decision variable set to one if $p_{i}\;or\;p_{j}=1$.

As can be noticed the dual issue will be solved for all points. This means that the computational effort will be higher than solving the original optimization problem. However, solving this optimization problem can be simplified by applying dynamic programming to eliminate the need to solve for the multiple points in the same path. The algorithm that combines the solutions for these two problems is shown in Algorithm 1. The restrictions in the algorithm are a key component of the solution. They ensure that tours are going to be eliminated. If the full path is known a priori, restrictions will only be added until an optimal solution is found. Otherwise, restrictions have to be changed to accommodate for new and visited nodes, and they will be updated with the proposed heuristic.
**Algorithm 1:** Optimization sequence
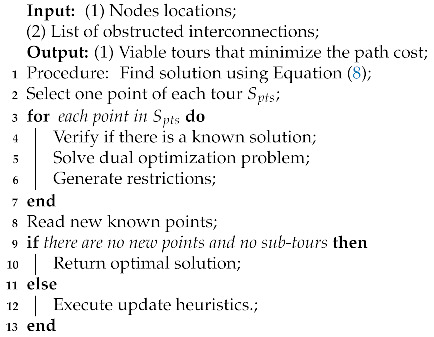


### 2.5. Heuristics Update

An essential step of the method is to manage the update of the information after the dual optimization problem. This step may have to deal with the different solutions and cuts found, as well as the mission execution information. During a mission execution, while visiting known points, a new one may be discovered. A strategy has to be applied in such a case, allowing continuous execution of the method without restarting from scratch. The strategy is to add restrictions to force that a given path was taken for the visited points.

Algorithm 2 was used to perform this step and was based on the ideas of other methods, such as Reference [48,49]. The main idea behind this algorithm is to allow new points to be added to the already established path solution with minimal disruption. A limitation that arises from this approach is that the points have to be added closer to the borders of the search space, which is the usual case for a CPP application, i.e., as the UAV explores the borders of the map, new areas are discovered and added to the map. In situations where points are added in random locations in the middle of the known space, the authors noted that the method efficiency is reduced significantly, and the computational time increases.
**Algorithm 2:** Update Heuristics
**Input**: (1) Nodes locations; (2) List of obstructed interconnections; (3) Solution found in step one; (4) New nodes to be added; **Output**: (1) Tours restrictions;**1** Procedure: Input new coordinates and generate new points $p_{new}$;**2**Generate list of new paths $p_{i,new}$ and $p_{new,i}$ for neighbors of $p_{new}$;**3**Find the nearest two neighbor of $p_{new}$;**4**Insert $p_{new}$ in the path;**5**Remove all tour restrictions associated to $p_{new}$ neighbors;**6**Update already visited points as restrictions to the solution Update restrictions matrices Update initial solution to new conditions;**7**Update restrictions matrices;**8**Update initial solution to new conditions.

In general, new points are expected to be added to the map borders while the UAV explores the area. This means that these situations, where points are added randomly to the map, are not likely to occur frequently. The authors have not exhausted these scenarios. However, a possible explanation is that adding a new point in already fully planned areas can significantly impact the optimal path. Thus, this may create a situation where many changes to the tour are required, and the previous solution is not a good initial answer.

## 3. Results and Discussions

Based on the concepts presented in Section 2, Proposed Methodology, this section presents the methods and strategies used for the simulations in Section 3.1, the obtained results for each proposed scenario, and a few discussions about them. A computer with the configuration i7 7700HQ with 16 GB of RAM and a GTX1060 6GB was used in the simulations. All simulations were executed five times. In Section 3.2, Optimization, the results were evaluated for the selected scenarios. In Section 3.3, Real Point Cloud, the results were evaluated for 3D reconstruction of a real scenario. In the end, Section 3.4, Fog-Cloud Computing, evaluates the algorithm execution in a distributed environment to determine its feasibility. The last experiment used a raspberry pi 4 with 4GB and the previous computer.

### 3.1. Methods

**Figure 12:**
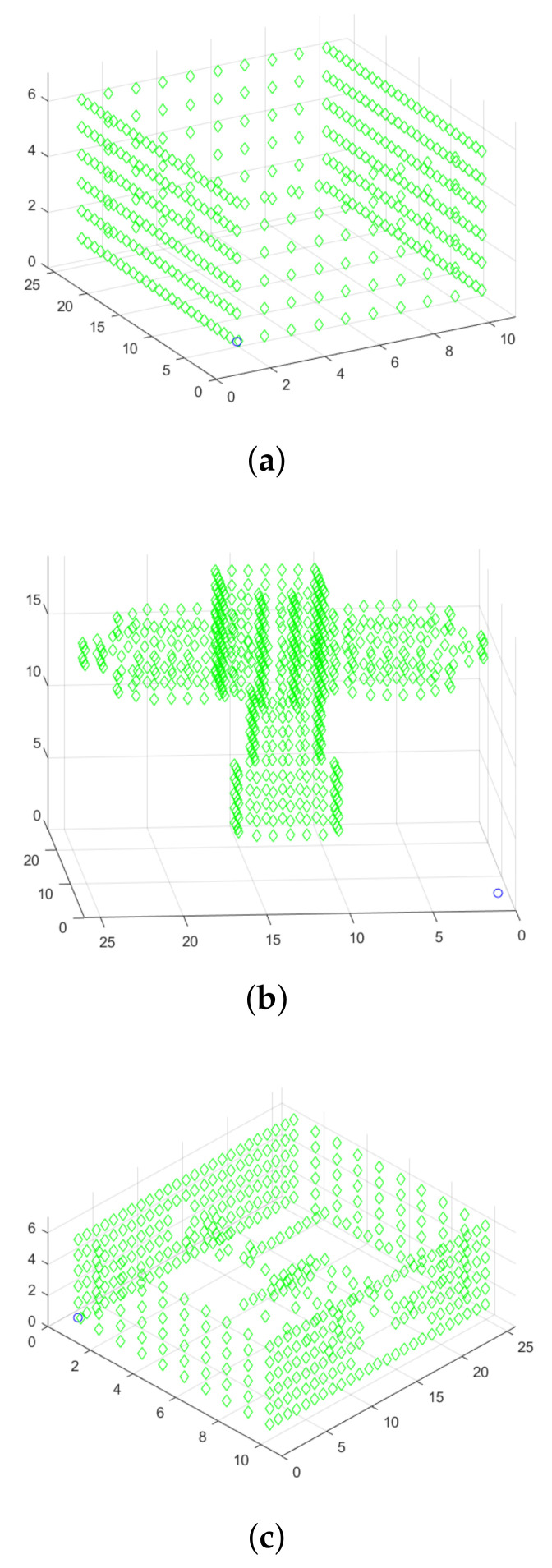
Path of the experiments. (**a**) Plain room. (**b**) Windmill. (**c**) Multi-room.

A few simulation scenarios were evaluated to show the planning effectiveness. First, two conditions are evaluated. In the first one, the UAV has full knowledge about the map state. In the second, only partial knowledge of a few blocks around the UAV position is known. For each of these conditions, the method was tested in three different scenarios. These scenarios were designed to reflect increasing levels of complexity. The first scene comprehends a simple plain room. The second one is an enclosed space with different rooms. The third contains a complex form similar to a windmill. Figure 12 illustrates these three scenarios.

In order to verify the 3DD-CPP method performance adequacy, a comparison with other methods in the literature is required. The planning performance for these three methods was compared with a Greedy Search and Genetic algorithm (GA). Both Greedy Search and GA were implemented by the authors to solve the issue based on [38,50]. The Greedy algorithm was implemented to select the closest waypoint to the current UAV position. The GA was implemented to reduce the total distance among cities [51]. Note that, when comparing different methods, it is important to establish the basis for comparison. Therefore, a few metrics were evaluated for all methods and scenarios, which are:

1. Computational time: Time between the start of the algorithm and a final path is found;
2. Total path length: Total path length using euclidean distance;
3. Energy consumption: Total energy used during navigation in the path selected;
4. Path complexity: Defining complexity is a complex task for general applications. However, a good measure is the number of times the UAV takes turns as a more linear path is usually smother. Here, a simplification is applied by calculating the relationship between the number of turns and the number of points.

### 3.2. Optimization

As described in the methodology, each method was executed in three different scenes (refered as “pr”: plain room, “mr”: multiple rooms, and “wm”: windmill) and two conditions (“kwn”: full map known and “ukwn”: unknown map). In each execution, the following variables were evaluated: (1) computational time (seconds), (2) total path length (meters), and (3) path complexity (dimensionless). The results are compiled in Table 3.

It is possible to note that the proposed method has a better path performance in all conditions when evaluating these results. However, it is also possible to highlight that computation times are high, which means that a proper CPU needs to be used to solve the desired paths. In the proposed cases, the Greedy solution is still feasible. However, this is more likely the result of symmetries present in the environment and may not be true for all cases. The GA results were obtained using an implementation based on the recent literature and provide a parameter for the obtained results. These results indicate that the 3DD-CPP is suitable for providing a very reasonable solution in a feasible computational time for a bounded number of points.

**Figure 13:**
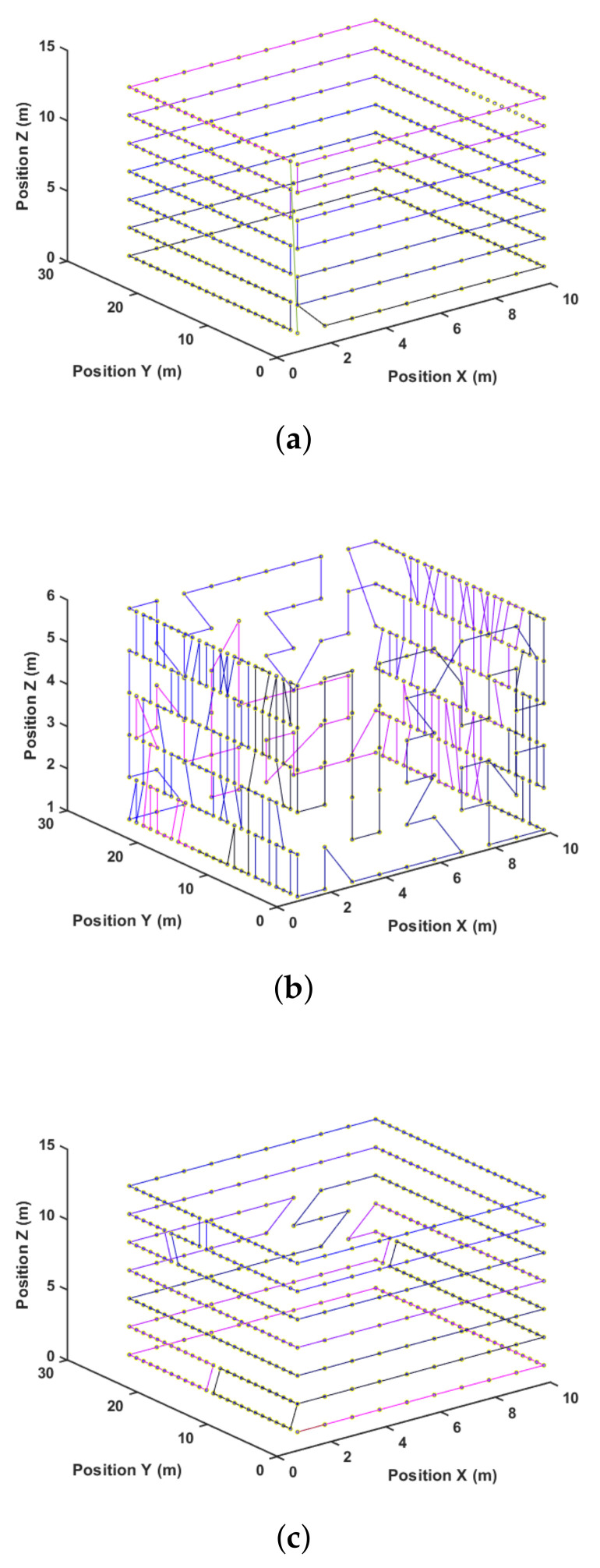
Path after solution for pr. (**a**) Greedy. (**b**) Genetic algorithm (GA). (**c**) Three-dimensional dynamic for Coverage Path Planning (3DD-CPP).

**Figure 14:**
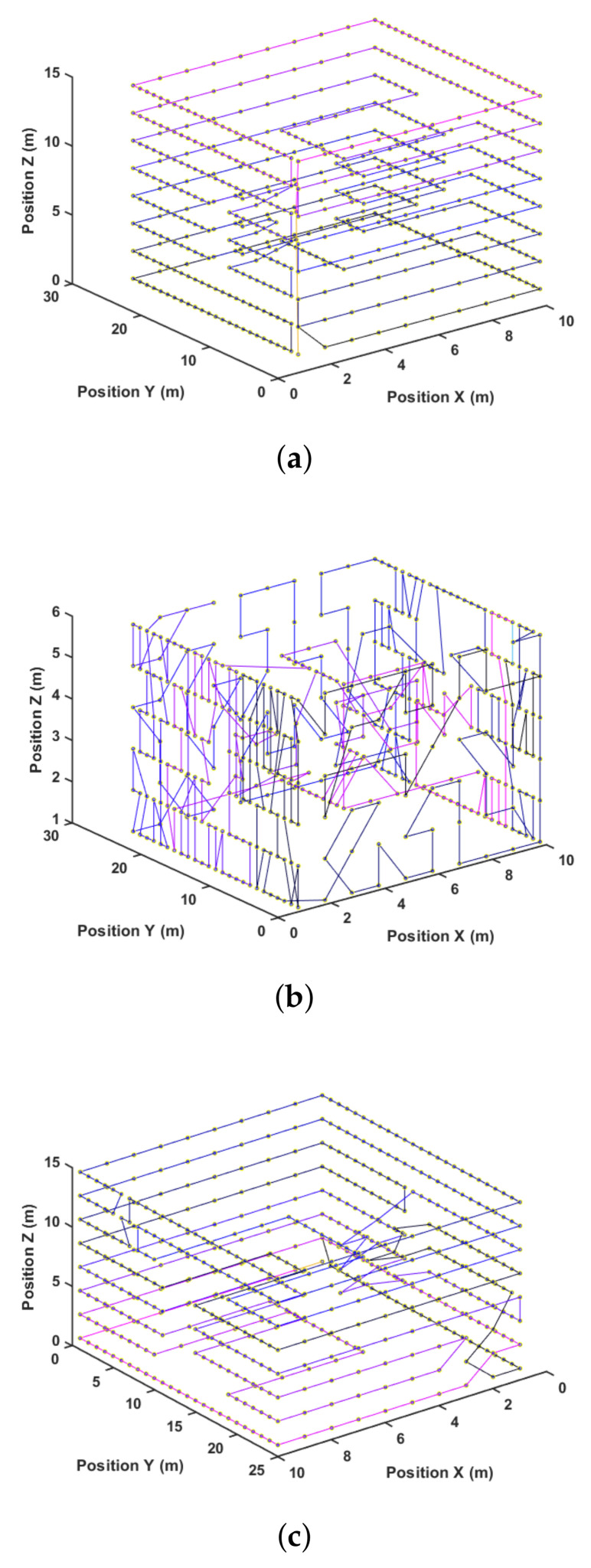
Path after solution for mr. (**a**) Greedy; (**b**) GA; (**c**) 3DD-CPP.

**Figure 15:**
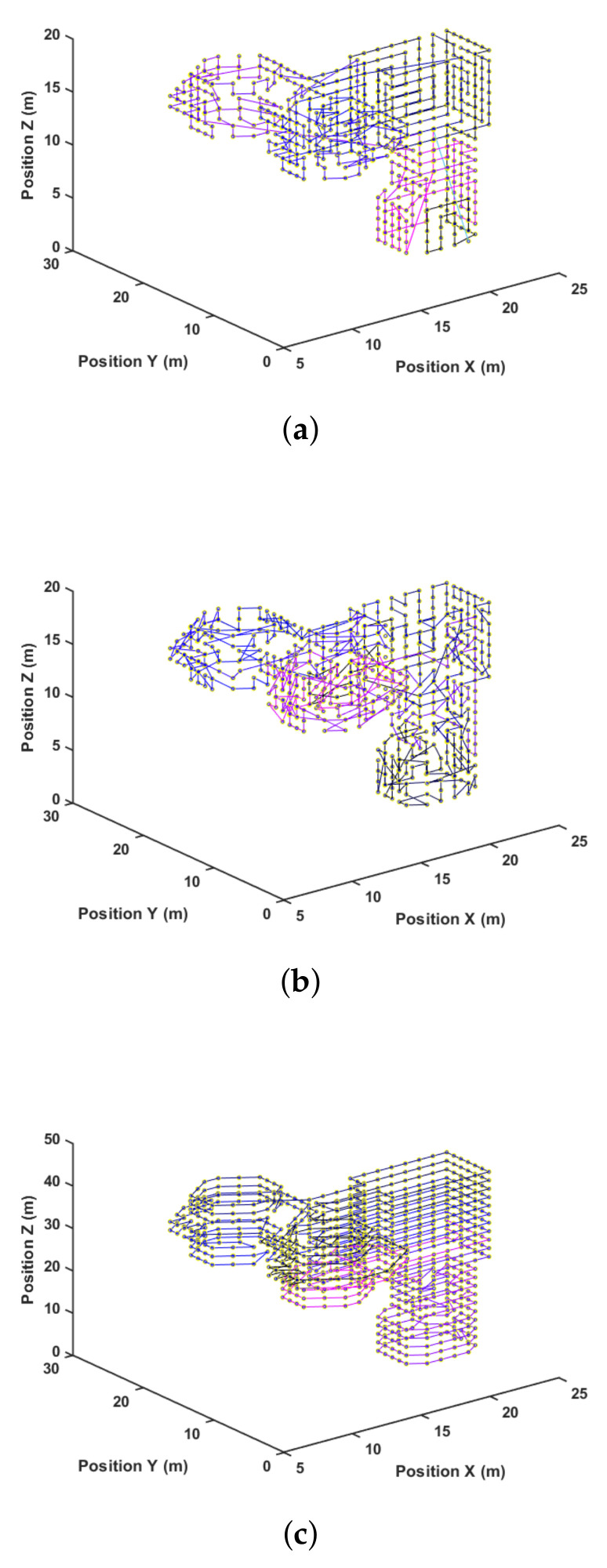
Path after solution for wm. (**a**) Greedy; (**b**) GA; (**c**) 3DD-CPP.

Another way to visualize these results is by looking at the paths generated for each method. Figure 13, Figure 14 and Figure 15 present the path representation for one solution obtained using each of the methods. Figure 13a, Figure 14a and Figure 15a show the solutions obtained by the Greedy Search. The used color lines range from black at the start to blue in the middle and purple at the end of the path to allow the user to grasp the movement. Symmetries in the environment and the waypoint distribution allow the Greedy algorithm to obtain a good solution. This may not be true in every environment, and the Greedy solution has no guarantee of finding any sub-optimal solution.

Figure 13b, Figure 14b and Figure 15b show the best solution obtained by the GA method after 25,000 steps and three different start attempts. This solution shows difficulties in capturing the environment’s symmetry, and this is also reflected in the quantitative result of Table 3. At last, Figure 13c, Figure 14c and Figure 15c present the proposed method result.

Among the results, an important variable to be evaluated is the number of turns, which is related to the energy consumption required to reduce speed and to perform the turn, increasing speed again [22]. This information is captured in the path complexity’s low relative values, and it is not captured in the proposed model. Thus, it represents a possible improvement for future works.

Note that the method captures the symmetries and finds an optimal path with a higher computational cost. It is also important to highlight that a fixed cost matrix was used to allow the Greedy Search and GA to present similar results. In real applications, the cost matrix would be calculated using the model in Section 2.3, Energy Cost Estimation. This means that these results are optimized for travel distance. However, energy consumption could be used only by using the proper cost matrix.

### 3.3. Real Point Cloud

**Figure 16:**
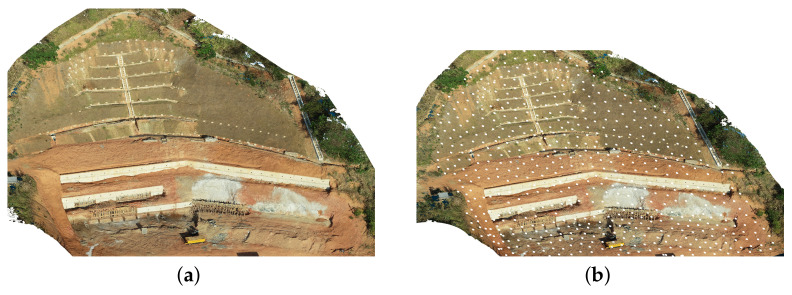
Inspection site: (**a**) 3D model; (**b**) waypoints.

From a previous inspection, it is possible to show the method’s potential in an offline scenario. Figure 16a shows a 3D model reconstructed from a inspection site. This model, in practical scenarios, can be obtained either from a laser scan, depth camera, or from previous inspections, as is the case for this work. The showed model has 150 m × 120 m of size and 32 m of height.

We can determine the distance from images and maximum distance from the inspected surface for a high amount of overlap between images and GSD. Using these parameters, one can sample waypoints in the surface using regular distances from each other. Figure 16b shows a representation for the waypoints obtained.

A high GSD value of 1 and 0.6 cm overlap was selected, resulting in 650 waypoints, each one representing a single picture to be taken. Note that this is an unusually high number of points for an area of this size. In regular applications, less than 150 pictures would be taken for the same area. However, the authors selected this configuration to allow better visualization of the method’s potential. In addition, note here that there is no optimization in pictures positions the positions of the pictures to allow them to form straight lines. In real-world scenarios, an optimization step would be used to ensure a path as smooth as possible. Despite not being usual when selecting inspection waypoints, these points make for a more difficult planning task.

**Figure 17:**
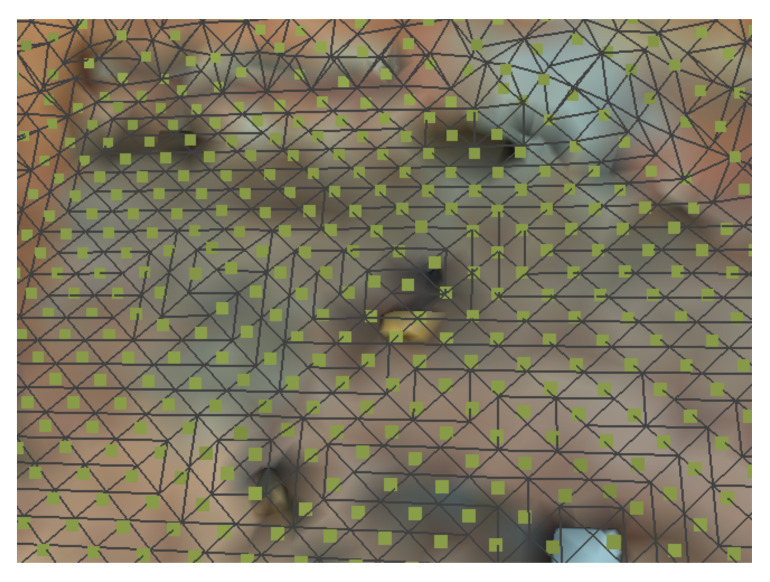
Example of mesh and sampling generated.

The waypoints are being generated by projecting the point cloud at a given distance from the object given by the minimum overlap required. Then, a mesh is estimated with the grid size stestimated from the minimum required overlap showed in the previous section. At the center of each face element in the mesh, we sample a point assuring that the number of points will be equidistant. The projected face element’s orientation is the reference for the surface orientation to be later used by the UAV when performing the inspection. Figure 17 shows the representation of the generated mesh.

Then, this information can be fed into the path planing algorithm in order to determine the optimal path. As in the previous results, the methods were compared with Greedy and GA algorithms to allow relative comparison.

The quantitative results in this environment are shown in Table 4. Note that the proposed method has the lowest length overall. In addition, the number of inequalities presented in the table is related to the complexity of the solution, i.e., how many restrictions had to be added to eliminate sub tours.

**Figure 18:**
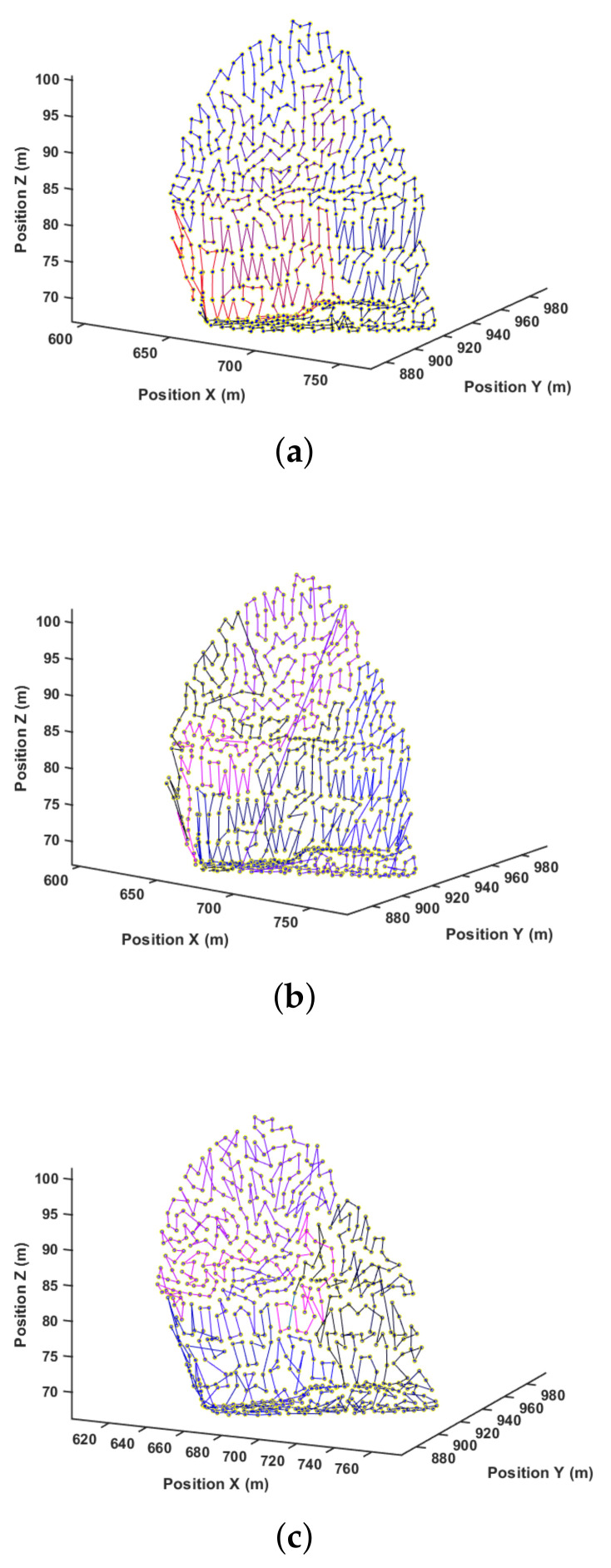
Path after solution. (**a**) Greedy; (**b**) GA; (**c**) 3DD-CPP.

Figure 18 shows the paths generated by each of the methods. The results showed the effectiveness of the 3DD-CPP method in this clustered and complex scenario. This is likely more similar to real-world mapping in contrast to planned missions. The computational time showed a similar trend to the previous results. However, the method is still feasible even for this large amount of points.

Despite the promising results, the authors understand that experimental tests will still have to be carried out in future works to bridge further the gap between the simulations and practice. Many considerations are made to allow method deployment. For example, the authors consider that the camera triggering process will be available, allowing trigger at the correct waypoints positions. The 3D environment model built online by the embedded computer using depth sensors will be accurate enough to enable method application. Those limitations, however, will also impact other techniques and will require further experimentation in future works.

### 3.4. Fog-Cloud Computing

Despite the viability of the obtained results, one can argue that the method is not suitable for real-world applications due to its computational cost. Indeed, the solution requires lots of CPU capability and would not be suitable for deployment in a low energy usage embedded ARM processor. In this sense, the authors also tested a scheme to verify the viability of the solution in a distributed environment where part of the computational load is executed onboard the UAV, and another part is at the fog. This is a suggested implementation, and it is not directly related to the proposed method. This section intends to show if the method is suitable to be used in UAVs, such as Intel Aero [52].

**Figure 19:**
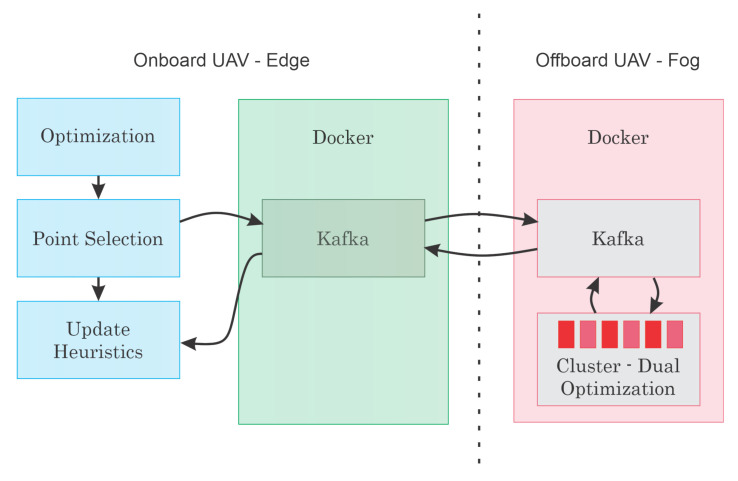
Edge-fog communication scheme simulation.

Figure 19 shows a representation of the proposed model. In this experiment, a docker container was deployed onboard a raspberry pi 4 with 4GB responsible for executing the main optimization step. A second docker container was used running in an intel i7 7700HQ with 16 GB of RAM and a Nvidia GTX 1060.

Inside the containers, a Kafka distribution is used to provide a structure for data communication. In Kafka, an input and an output set of topics were deployed. Each Offboard cluster can now read the points to be optimized using the dual problem and calculate the respective cuts to be used in the next iteration of the primary problem. At each cycle, the solution will step towards the optimal value.

The code was executed for the same problem from Section 3.2, Optimization Results. Table 5 shows the obtained results. Note that the total processing time increased slightly. This is likely associated with the limited capability at the edge node. However, the processing time still makes it feasible for the online deployment of the method.

## 4. Conclusions and Future Work

This research addresses the utilization of mathematical models to perform path planning for CPP applications. The novelty of the 3DD-CPP is the combination of heuristics to allow the technique’s deployment either online or offline. In this research, a model to allow edge-fog implementation is also evaluated using simulated data. The results showed that the proposed methodology is viable and effective.

Results also showed that the proposed heuristics are capable of enabling re-optimization, allowing execution in unknown environments. Despite the method’s good results, the execution online is still somewhat limited to a certain number of points. This may limit the deployment in very dynamic environments and open space for further dynamic obstacle avoidance developments. The method is also evaluated for a high number of points using real data. The result showed that the method is feasible for practical applications.

A shortcoming of methods that rely on high computational power, such as this one, is the limited resources onboard UAVs. However, the method’s main characteristic is that the dual optimization issues are not interdependent. Therefore, they can be solved in parallel. To this end, the results also indicated that the parallel execution of the method in an edge-fog system is viable, producing little increase in solution time.

In future works, the model should be updated to enable multiple agents to operate at the same time. Practical experiments will also be carried out by combining visual odometry methods building the local map and allowing method deployment. The practical experiments will be carried out using techniques, such as Reference [53], in combination with depth cameras to enable the authors to build the point cloud maps online. This effectively will enable authors to fill the gap of modeling the 3D geometry of the environment required for the proposed method operation. The authors also intend to provide a second heuristics to consider obstacle avoidance for dynamic objects that move close to the UAV space. The authors also intend to include path complexity as a variable for optimization in the model.

## Figures and Tables

**Table 1:** Literature background comparison.

Paper	Publication	Year	Citations (Scholar)	1.	2.	3.	4.	5.	6.
3DD-CPP	-	-	-	Mathematical	✓	✓	✓	Optimal if full path is known	✓
Di Franco [32]	Conference	2015	203	Heuristic	-	-	✓	Asymptotically	-
Bircher et al. [33]	Conference	2015	145	Mathematical	-	-	✓	Optimal if full path is known	-
Torres et al. [34]	Journal	2016	109						-
Han et al. [35]	Journal	2017	74	Heuristic	✓	-	-	Sub-optimal	-
Janson et al. [36]	Journal	2017	68	Heuristic	-	-	✓	Assintotically	-
Cabreira et al. [37]	Journal	2018	34	Heuristic	✓	-	×	Sub-optimal	-
Glorieux et al. [17]	Journal	2020	5	Heuristic	-	-	✓	Sub-optimal	-
Majeed and Lee [22]	Journal	2019	8	Heuristic	✓	-	✓	optimal	-

**Table 2:** Motor characteristics.

Motor T-Motor 3515 for 3 Cell Batery and 15 Inch Propeller		
**Current**	**Power**	**Thrust**
3.4 A	75.5 W	0.780
6.3 A	139.9 W	1.180
8.5 A	188.7 W	1.480
11.4 A	253.1 W	1.800
13.7 A	304.1 W	2.010

**Table 3:** Quantitative results from the evaluated methods: (1.) Computational time (s), (2.) total path length (m), (3.) energy consumption (w), (4.) path complexity.

Algorithm	Scene	Condition	Parameter			
			1.	2.	3.	4.
3DD-CPP	pr	kwn	92 s	477.0 m	25	0.113
		ukwn	-	505.2 m	60	0.255
	mr	kwn	118 s	606.17 m	44	0.156
		ukwn	-	812.17 m	121	0.321
	wm	kwn	135 s	911.1 m	170	0.421
		ukwn	-	1305.55 m	243	0.493
Greedy	pr	Indifferent	2 s	480.3 m	25	0.076
	mr	Indifferent	2 s	620.8 m	61	0.137
	wm	Indifferent	2 s	995.6 m	195	0.400
GA	pr	kwn	34 s	520.4 m	145	0.781
	mr	kwn	32 s	1239.0 m	202	0.351
	wm	kwn	36 s	2147.0 m	601	0.870

**Table 4:** Quantitative results in real environment waypoints.

Method	Length	Number of Inequalities	Computation Time
3DD-CPP	2821 m	1585	113 s
Greedy	3230 m	-	5 s
GA	3588 m	-	65 s

**Table 5:** Results from distributed execution test.

Scene	Parameter		
	Processing Time ` at Edge	Processing Time at Fog	Network Average Ping
pr	32 s	84 s	10 ms
mr	37 s	102 s	10 ms
wm	38 s	112 s	10 ms
